# Individuality and Togetherness in Joint Improvised Motion

**DOI:** 10.1371/journal.pone.0087213

**Published:** 2014-02-12

**Authors:** Yuval Hart, Lior Noy, Rinat Feniger-Schaal, Avraham E. Mayo, Uri Alon

**Affiliations:** 1 Department of Molecular Cell Biology, Weizmann Institute of Science, Rehovot, Israel; 2 The Theatre Lab, Weizmann Institute of Science, Rehovot, Israel; 3 Graduate School of Creative Arts Therapies, The Center for the Study of Child Development, Haifa University, Haifa, Israel; University of Maribor, Slovenia

## Abstract

Actors, dancers and musicians that improvise together report special moments of togetherness: high performance and synchrony, seemingly without a leader and a follower. Togetherness seems to conflict with individuality- the idiosyncratic character of each person's performance. To understand the relation of individuality and togetherness, we employed the mirror game paradigm in which two players are asked to mirror each other and create interesting synchronized motion, with and without a designated leader. The mirror game enables quantitative characterization of moments of togetherness in which complex motion is generated with high synchrony. We find that each person as a leader does basic strokes of motion with a characteristic signature, in terms of the shape of their velocity profile between two stopping events. In moments of togetherness both players change their signature to a universal stroke shape. This universal velocity profile resembles a half-period of a sine wave, and is therefore symmetric and maximally smooth. Thus, instead of converging to an intermediate motion signature, or having one player dominate, players seem to shift their basic motion signatures to a shape that is altogether different from their individually preferred shapes; the resulting motion may be easier to predict and to agree on. The players then build complex motion by using such smooth elementary strokes.

## Introduction

Studies on improvisation in music and motion have mostly focused on a single improviser [1]–[3]. When people improvise together, special phenomena can arise. Experienced musicians, actors and dancers that improvise together report special moments of high performance and synchrony [4]. These are moments of creativity that arise out of the interaction between people, seemingly without a leader and a follower. As musicians often describe it, ‘The music played us’. These moments can be defined as moments of togetherness. This may relate to concepts such as ‘being in the zone’ in theatre and sports, described as “a state of unselfconscious awareness in which every individual action seems to be the right one and the group works with apparently perfect synchronicity” [5]. In anthropology, togetherness relates to communitas [6] and interpersonal synchrony in meaningful rituals [7], and in psychology it may relate to the concept of group flow [8], [9] and dyadic states in parent-infant interaction [10].

**Figure 1:**
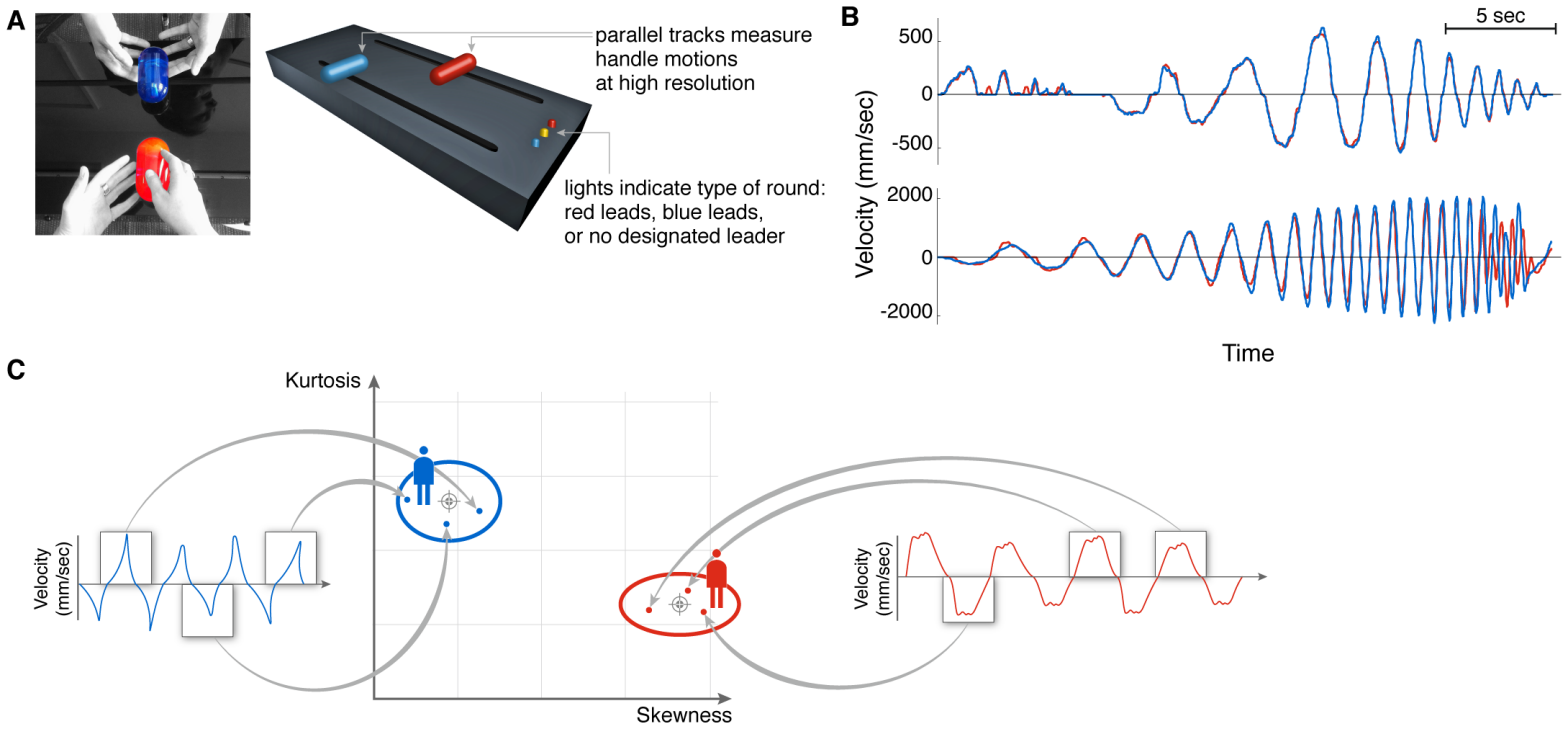
Joint improvised motion in the mirror game was analyzed in terms of elementary motion events called segments. (A) In the one dimensional mirror game players move handles along parallel tracks, and motion is tracked. Lights indicate type of round: red leads, blue leads or no designated leader. (B) Examples of velocity traces from two different games. Red trace marks the red player and blue trace marks the blue player. All traces are taken from rounds with no designated leader. Notice the high synchronization of the motion in both cases and its relative complexity. (C) A motion segment is defined as the velocity trace between two consecutive zero velocity points. The shape of segment velocity traces is characterized by two parameters: skewness – the shift to the left or right, and kurtosis – the relative weight on the curve ‘shoulders’. Throughout the paper each segment is described as a point in this two-dimensional low-level motion parameter plane. The segment characteristics of each player are described by an ellipse whose center is the mean and its axes are the standard deviation (error bars) of the skewness and kurtosis values.

Recently, building on the growing field of joint action research [11]–[18], a paradigm to experimentally study togetherness was presented [19]. This paradigm is based on the mirror game, a theatre exercise whose purpose is to help actors experience moments of togetherness [20], [21]. In the experiment, players were told to create interesting and synchronized motion as they mirrored each other moving handles along parallel tracks (Fig. 1A), with and without a designated leader. When a player was designated as leader and the other as follower, the leader made smooth motion, whereas the follower showed a characteristic 2–3 Hz oscillation around the leader's confident trajectory. Similar zero-lag oscillations were previously observed when human subjects manually tracked a visual target, and were interpreted as an indicator for a reactive response mechanism [22]. This oscillation, termed jitter, can thus be used as a mark of followership. When there was no designated leader, expert improvisers generated complex motion together. About 15% of the time, they generated especially synchronized and complex motion, in which neither player showed jitter: Both players showed confident, smooth motion characteristic of two leaders. This co-confident motion was suggested to be an example of togetherness. Recent works have used similar interpersonal motor mimicry paradigms to study other facets of social interaction and togetherness [16], [18], [23], [24].

One question raised by the phenomenon of togetherness is its relation to individuality. Each person presumably has idiosyncratic, individual character to their performance, whereas togetherness implies unity in which performance of individuals merges into a synchronized whole. Is individuality lost in togetherness? What is the nature of performance in togetherness? For example, is it a weighted average or a blend of the two individual performances or something altogether different?

Researchers have identified individual differences in a large number of motor variables, including for example reaction time, speed and preferred frequency of arm movement and multi-limb coordination [25]–[27]. People swinging pendulums together converge to a frequency which is intermediate between their individually preferred frequencies [28], [29]. To our knowledge, there have been no quantitative studies on individual performance in joint improvisation.

Here, we address the question of individuality and togetherness, using the mirror game. We study each player's individual characteristics in making basic strokes of motion, and find that people show individual signatures in motion space. We then ask what happens to these motion signatures as two players perform co-confident motion, creating synchronized complex motion together.

## Methods

### Ethics Statement

The Institutional Review Board (IRB) at the University of Haifa approved the described experiments, including the written consent procedure (approval number 086/13). All the participants provided their written informed consent to participate in the study.

### Setup

A customized device measured the linear motion of two handles at 50 Hz, with spatial accuracy of 1 mm (Fig. 1A and 1B). A set of lights indicated the type of round (blue leads, red leads, or no designated leader). Players were instructed to produce mirror-like motion together that is synchronized and interesting, with or without a designated leader. See details in [19].

### Participants and Procedure

The dataset contains three sets of experiments with different players. Experiment 1 had nine pairs of experienced improvisers (actors and musicians with over ten years of experience in group improvisation) as described in [19]. Experiments 2 and 3 had a single experienced improviser (Exp2: TI, male, aged 32, Exp3: ET, female, aged 27), each playing with 23 different novices of the same gender. The repeating expert was always the red player. Each game in Experiment 2 and Experiment 3 had three rounds (blue player leads, red player leads and no designated leader) of three minutes each. A one minute practice round preceded each game. The games in experiments 2 and 3 began with the novice as leader, followed by the expert as leader, followed by a round with no designated leader or follower, so that the novice's leader motion would not be primed by the repeating player motion. We also analyzed eight games with novice players as a control (see SI of ref [19], and SI, Fig. S10 in File S1). The motion data is available in http://www.weizmann.ac.il/mcb/UriAlon/download/downloadable-data.

### Preprocessing

Segments were defined as periods of motion between two zero velocity events. We removed segments shorter than 0.2 s or longer than 8 s, and segments with less than 3 cm displacement. In the current experiments 18% of the total motion time was removed.

We previously found a typical 2–3 Hz jitter pattern in the motion of the follower in the mirror game [19]. We performed a correlation analysis on the leader-follower motion and find that correlation peaks at zero lag (see SI, Fig. S12 in File S1). This is because the follower's jitter motion weaves around the leader's motion (with a 2–3 Hz period). Thus, the follower is sometimes ahead and sometimes behind the leader.

We therefore automatically detect the 2–3 Hz jitter motion, and consider highly synchronized periods without jitter as co-confident (CC) motion periods. CC periods were defined [19] as periods of non-zero velocity longer than 2 s in which the Fourier rms power in the 2–3 Hz band of the difference between the players' velocities was less than 10% of mean velocity rms, and less than 40% of mean velocity rms of mean motion at frequencies above 2 Hz.

We also used an alternative definition for co-confident motion, using the notion that togetherness results in highly synchronized motion. In the alternative definition, we considered motion in which the rms relative velocity error between players was smaller than 35%, and the difference in stopping times was less than 80 ms.

A segment was labeled as a CC segment if at least 80% of its sample points lay in CC periods. We excluded from the analysis games that did not contain at least 15 CC segments for each player.

### Data Analysis

For each segment we computed four shape characteristics: center of mass, 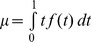, variance, 
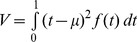
, skewness, 
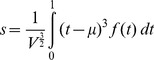
 and kurtosis, 
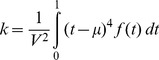
. These are the moments of f(t), the recorded velocity values measured between the start and end of each segment, with time normalized between zero and one, and velocity normalized by its integral over each segment 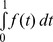. The distribution of segments of a given player in the skewness-kurtosis plane can be described by an ellipse, whose center is at the trimmed mean and ellipse axes are the trimmed standard deviation of the segments features. The ellipses height and width represent the errors bars of the motion data (see SI, Fig. S8 in File S1). Trimmed mean and standard deviation were calculated by removing the top and bottom 10 percent quantiles of data values and then calculating the mean and the standard deviation, respectively. We compared the difference of mean skewness and kurtosis values for different subjects using t-tests. Because data was often not normally distributed, we also used Mann-Whitney tests [30]. Multiple testing errors were controlled for by using the Benjamini-Hochberg false detection rate (FDR) method [31]). Other statistical tests (Kolmogorov-Smirnov, Anderson-Darling and Cramer-von Mises) are described in the SI, File S1.

We tested whether the two definitions for CC motion described above are affected by skewness-kurtosis features, using computer generated data with high synchrony between the simulated players and different values of skewness and kurtosis. We find that this joint simulated motion is detected as CC motion, regardless of its skewness and kurtosis values (see SI, Fig. S11 in File S1).

## Results

### We analyzed the basic elements of motion in the mirror game: segments, defined as periods between zero velocity

We analyzed 55 different games (nine from ref [19] and 46 from the present experiments) with 60 different players. Because our interest in this study is in periods of togetherness in the mirror game, we excluded from the analysis games that did not have at least 15 distinct motion segments displaying co-confident motion (defined as in ref [19] as periods of motion with high synchrony and low jitter) in rounds with no designated leader.

The remaining dataset includes 30 games (six from ref [19] and 24 from the present experiments), with 33 different players. The games from Experiment 1 included six expert-expert pairs (one female-female, four female-male, one male-male). The games from Experiment 2 included 16 male expert-novice pairs. The games from Experiment 3 included eight female expert-novice pairs. We find no correlation of player gender with the effects reported here (SI, Table S6 and Fig. S9 in File S1).

In these games, co-confident motion averaged 17±2% of the duration of the rounds with no designated leader. The co-confident fraction is similar in the two datasets (the six games analyzed in ref [19] had 16% and the 24 new games in this study had 18% co-confident motion).

To analyze the basic elements of motion, we divided the motion of each player into segments between zero velocity events (see Methods for details). Segments averaged 0.8 s in duration (standard deviation = 0.7 s, median = 0.5 s). The resulting dataset included 35660 segments from the 30 games.

We next classified the shape of the segment velocity traces. We normalized the velocity trace of each segment by its entire mass, and normalized the time axis of each segment between zero and one. We calculated the first four moments of the velocity trace. The first moment, *μ*, describes the center of mass of the curve, and the second moment describes its variance, *V*. The third and fourth moments are called skewness and kurtosis. Skewness indicates a shift of the curve to the right (negative skewness) or left (positive skewness). Kurtosis measures the flatness of the curve around its peak, the ‘shoulders’ of the curve (see Methods). Fig. 1C shows skewness and kurtosis of example velocity traces. These measures capture the shape of the segment curve, and are not significantly affected by the amplitude or frequency of the motion in the dataset (see SI, Table S1 in File S1). We also analyzed the motion using Fourier components, and find similar qualitative conclusions (see SI, Table S2 and Fig. S1 in File S1).

### Each player has individuality: distinct motion characteristics

We compared the motion characteristics of each player during rounds when that player was designated as leader. We did not include the two expert players with repeated games (their motion shape is described below). Thus, the comparisons are mostly between two players in different games. We used student t-tests to compare the means of different players (and, because data is not always normally distributed, we also used Mann-Whitney (MW) tests, with similar results). We find that players have similar first and second moments of their segment velocity curves (see SI, Fig. S2 in File S1). However, the skewness and kurtosis reveal players' individuality.

We find that 79% of the comparisons between pairs of players are different for skewness or kurtosis (mean t = 5.2, mean p = 0.003, all p<0.03). We controlled for multiple hypothesis testing using the Benjamini-Hochberg FDR procedure with error set to 0.05 [33] (85% in Mann-Whitney test, and see SI Fig. S3 and Table S3 in File S1, for details and other statistical tests).

**Figure 2:**
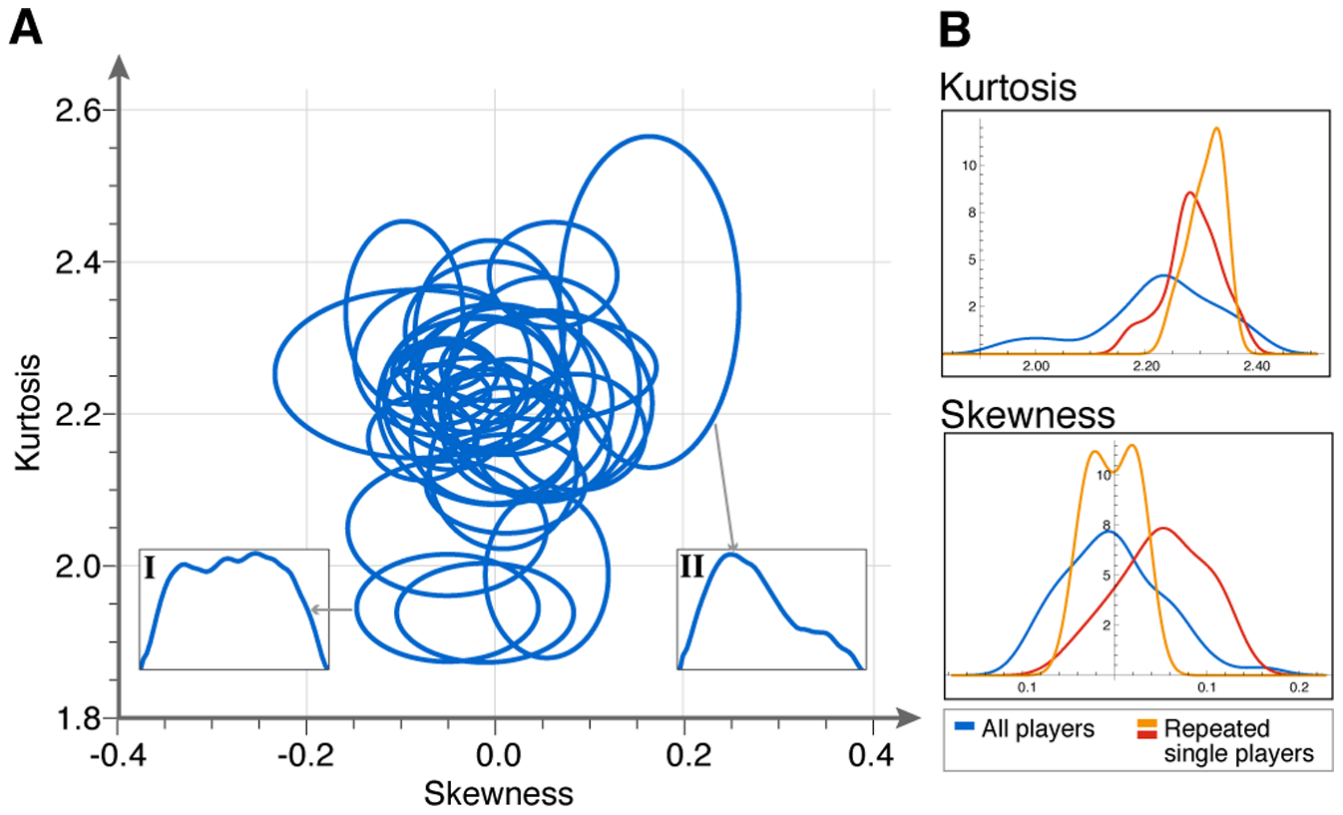
Players show individual signature in the shapes of their segment velocity traces. (A) The motion of each player while playing as a leader is represented by an ellipse, which represents one standard deviation around the mean of all segments by that player in one game (between 47 to 392 segments/game, median = 170 segments/game). Insets are examples of velocity segments. (B) The distribution of different players' mean kurtosis and skewness values as leaders (blue), and of two expert players who played multiple games (16 and 8 games, red and orange curves).

To visualize the player's individuality, we plotted the segments on the skewness-kurtosis plane. The motion of each player corresponds to a cloud of points on this plane. We plotted for each player, ellipses that represent the standard deviations (error bars) around the mean. It is evident that each player occupies a different region of this plane (Fig. 2). The mean of each player is separated from the mean of other players by up to four standard deviations.

Similarly, comparing the two players in each game shows that their motion characteristics significantly differ in 70% of the games by t-test (with p<0.02, mean t = 4.7, mean p = 0.003, FDR of 0.05) and 80% by Mann-Whitney test (see SI, Table S4 in File S1). We find no correlation between use of the red or blue handle in the mirror-game setup and the motion characteristics (see SI, Table S5 in File S1).

We also tested how constant across games are the motion characteristics of a given player. We find that the two repeating players in our dataset, who played with 8 or 16 other players, showed motion characteristics which are quite constant across games (mean varies between games by 1%, standard deviation varies by 10% to 25%, Fig. 2). These repeating players are also different from each other. This suggests that, at least for these repeating experts, individuality remains approximately constant across games. If we take the variation in the same player across games as a measure of the repeatability of the experiment, we find that the differences between individual players are on average four-fold larger than the variation in the same player across games.

### In togetherness periods (co-confident motion), motion characteristics are universal across players

We next analyzed periods of co-confident motion in rounds where there was no designated leader or follower. Co-confident (CC) motion was defined using the criterion of our previous study [19]. This yielded a total of 5326 co-confident segments (14.9% of segments). The alternative definition of co-confident motion, where togetherness is defined as highly synchronized motion (see Methods), resulted in 5896 highly synchronized segments (16.5% of all segments), with a 44.2% overlap with CC segments defined above. Both definitions gave the same qualitative conclusions, and hereafter we use the definition of Ref. [19] (see SI, Fig. S7 in File S1, for more details).

**Figure 3:**
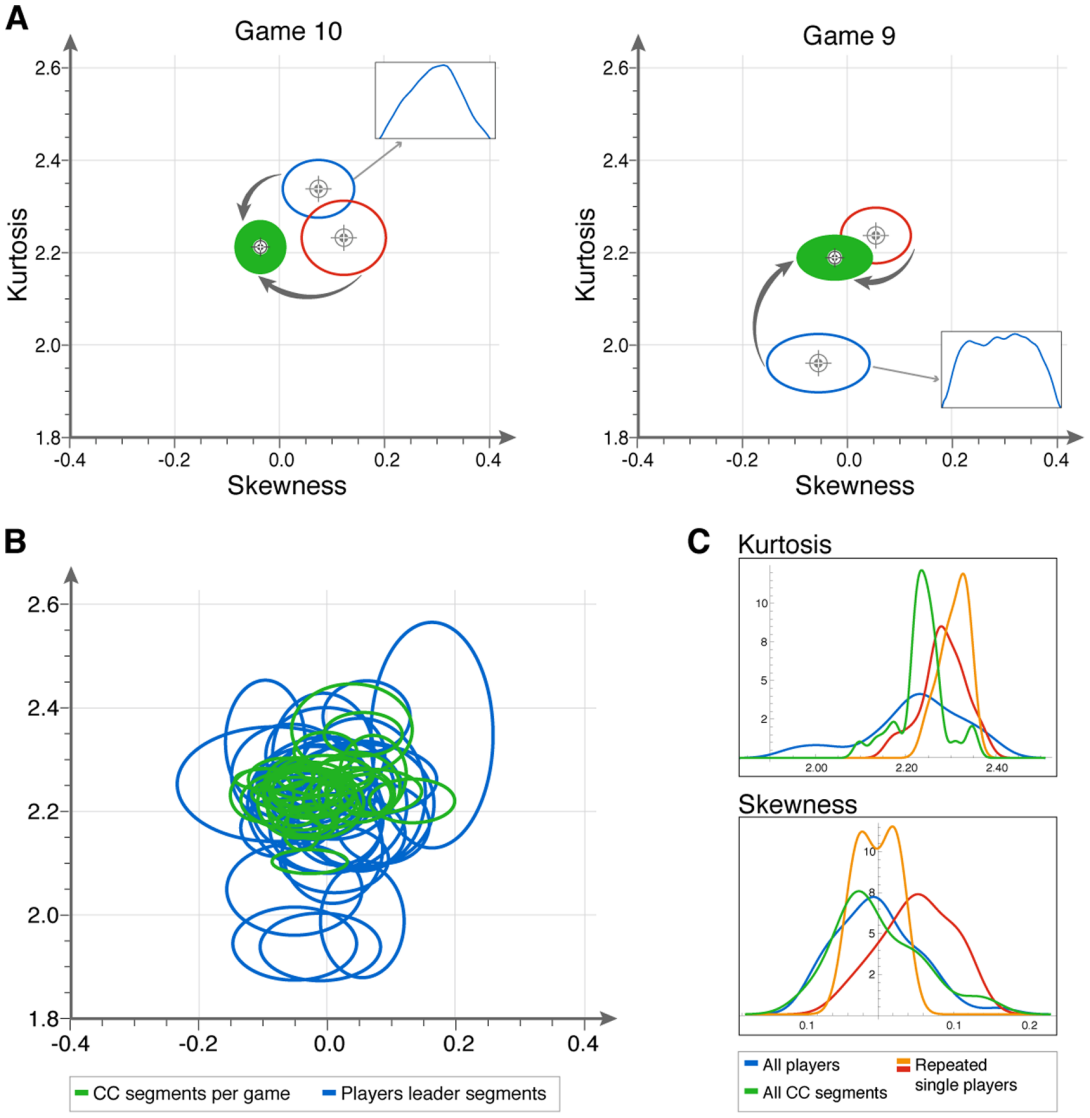
In co-confident motion, all players show a universal shape in their segments. (A) Examples of two games in which players have individual segment characteristics when they lead (blue and red ellipses), and show a distinct segment shape in CC motion (full green ellipses). (B) Despite the variability in player signatures as leaders (blue ellipses), all players converge on a similar region of segment shape space during CC motion (green ellipses). (C) The distribution of players' mean kurtosis and skewness values as leaders (blue) and in CC motion (green). Also shown are the leader characteristics of the two expert players who played multiple games (repeating players, red and orange).

We find that co-confident motion of different players in different games has very similar characteristics (Fig. 3A and 3B). Different players CC motion falls in a small region around skewness 0±0.04 and kurtosis 2.2±0.02. We find that the standard deviation of the CC motion of 30 different players is similar to the standard deviation of the same player playing repeated games (see Fig. 3C). Moreover, the standard deviation of kurtosis values in CC motion of the different players is three-fold smaller than the maximum difference between two players' motion playing as leaders.

We compared players' motion as leaders to their motion in CC periods in joint-improvisation rounds. We find that in 15 games out of our dataset of 30 games (50%), players' motion signature as leaders was significantly different from their motion in CC periods (t-test mean t = 4.8, p<0.006, mean p = 0.003, FDR set to 0.05, Mann-Whitney tests resulted in 60%, see also SI, Table S7 and Fig. S8 in File S1).

We term the corresponding region of skewness-kurtosis plane the ‘universal CC region’. Even players with very different segment characteristics as leaders converge to the universal CC region (Fig. 3A). Moreover, two players who happen to match in their idiosyncratic segment shapes, change to the universal shape (Fig. 3A, left panel) when they reach co-confident motion.

We also analyzed games where both players were novices. The probability for a co-confident segment was almost three-fold lower than in games with an expert (see SI, File S1). However, the co-confident motion in these games also converged to the same universal CC region (see SI, Fig. S10 in File S1). The mean motion characteristics of experts and novices as leaders were not significantly different (t-test, t = 2.9, p = 0.26).

### The segments in the universal co-confident region resemble a half-period of a sine wave

**Figure 4:**
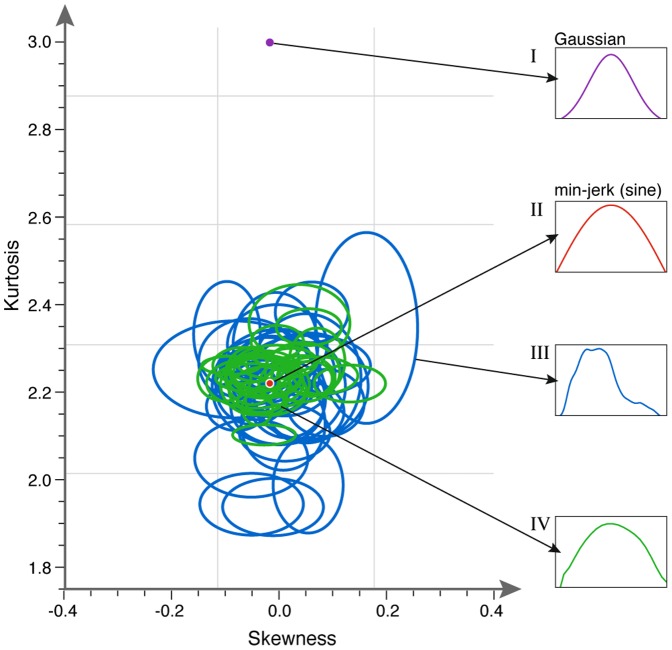
The universal co-confident segments are symmetric and smooth. Co-confident segments (green ellipses) cluster around kurtosis and skewness values similar to the minimal jerk solution for periodic motion (the trace which minimizes the integral over the acceleration change squared, resembles a half-sine wave). The characteristic of the half-sine wave lie in the center of the co-confident region in segment shape space. For comparison, a Gaussian trace, with kurtosis computed as 3, is shown far from the observed motion. Insets: pure Gaussian and half-sine traces and two examples of traces from the dataset.

The universal co-confident region describes segments which are symmetric (near zero skewness) and have relatively low kurtosis of 2.2±0.02 (Fig. 4). The center of the region is a segment whose shape resembles a half-period of a sine wave. This is nearly identical to the solution of periodic motion with minimal changes in acceleration (minimal jerk defined by 
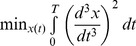
), which characterizes natural motion in experiments in which people perform point-to-point curvilinear motion, as described by Flash and Hogan [32]–[34]. These motions are thus, in a sense, maximally smooth (see SI, Fig. S4 in File S1). More detailed analysis of the CC region, including harmonic decomposition analysis, is provided in the SI (Fig. S2 in File S1). In addition, the CC segments are more distributed around a typical spatial length (24±2 cm) than segments in leader motion (36±7 cm) (see SI, Fig. S5 and S6 in File S1).

## Discussion

This study addressed the question of how individuality relates to togetherness, in the joint improvisation of motion. We studied the basic elements of motion, namely segments of movement between stopping events, in the mirror game paradigm. Players show individual, idiosyncratic motion characteristics when they act as leaders. A player shows the same individual segment characteristics in different games. Thus each player's basic motions constitute a personal signature that occupies a distinct region of shape space. In contrast, when players improvise together in co-confident motion- synchronized motion with no designated leader or follower- their segment shapes are restricted to a small and universal region of the shape space. The co-confident segments are symmetric and smooth, resembling a half-period of a sine wave. The players build complex motion together out of these smooth elementary motion strokes.

**Figure 5:**
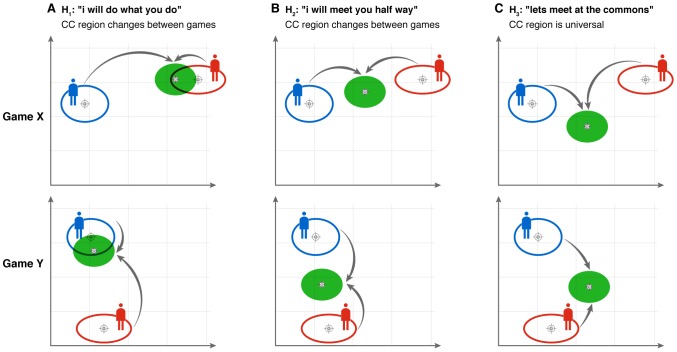
Players meet at a universal region of motion space when in togetherness, instead of meeting at their mean motion. Schematics of three possible hypotheses: (A) Hypothesis One: One player mimics the other players' segment signature during co-confident motion. (B) Hypothesis Two: Both players tune their signatures and meet, during co-confident motion, at an intermediate position in segment shape space. Meeting region is different for each game and depends on both players' signatures. (C) Hypothesis Three: All players tune their signatures to meet at a universal region of the segment shape space. Co-confident motion region is common to all games. This describes the present findings.

Given that players show individual motion waveforms (as reflected in their skewness-kurtosis values) what could be the expected waveform during periods of togetherness? One reasonable hypothesis is that players would produce intermediate waveforms. This hypothesis is analogous to the ‘magnet effect’ in physiology [35], in which coupled oscillators with different intrinsic frequencies tend to converge on an intermediate frequency [28]–[31], [37] (an imperfect analogy because it concerns frequency and not waveform). Another possible hypothesis is that one player would dominate, in the sense that the second player would take on the characteristics of the first. However, in the mirror game players do not meet at an intermediate segment shape (Fig. 5B), nor does one or the other dominate (Fig. 5A). Instead, all players move to a particular type of segment shape when they attain co-confident motion (Fig. 5C). Even two players who happen to match in their idiosyncratic segment shapes, change to the universal shape (Fig. 3A) when they reach co-confident motion. Co-confident motion is not only smoother than leader motion. It shows a limited range of segment shapes: the CC segments are similar to half-periods of a sine wave (Fig. 3B, 3C and 4).

The segments in the universal CC region are therefore maximally smooth and symmetric. We hypothesize that smooth and symmetric traces are essential for co-confident motion because they are easy to predict; one can extrapolate with relative confidence how the segment will end based on its beginning. Thus, togetherness may be linked with a specific type of elementary motion- motion that makes it easy for players to match predictions for future movement. This interpretation is in line with recent studies showing that co-actors tune their motion to be more predictable when engaging in dyadic interactions in simple joint-action tasks, a strategy that is presumably used to enhance coordination [36]–[38].

This study focused on the basic elements of motion- strokes of movement between two stopping events. We did not study the nature of the movement formed out of these strokes. It's as if we studied the way people wrote letters, not which words they wrote. The complexity of motion during co-confident (togetherness) periods has been found to be high [19]: players performed motions with sudden changes in tempo and shape, and with crescendos and diminuendos in amplitude and frequency. Thus, togetherness does not necessarily entail simple or periodic motions. Future work can examine whether players have idiosyncratic usage of motion ‘words’ composed of multiple segments. Such studies will require much more data than the current study.

In our previous study of the mirror game [19], we found that pairs of expert improvisers showed co-confident motion, whereas pairs of novices showed co-confident motion much more rarely. In experiments 2 and 3 of the present study, an expert played with a novice player. A similar level of co-confident motion was found as in two-expert games. Thus, one expert seems to be enough to reach togetherness in the mirror game.

Each of the two expert players in experiment 2 and 3 produced approximately constant segment motion characteristics across games. This suggests that individuality remains approximately constant across games. It would be interesting to check this also for novice players by having the same novice play several games against several different players (e.g. experts), allowing a test of consistency among individual novices. An interesting question that arises is how many games are needed before a novice gains expertise in the mirror game. Although we define experts as people with ten years in improvisation, it may take much less time to gain expertise in the mirror game.

A future avenue of research can study the neural mechanisms related to the behavioral results reported here, in accordance with a number of recent studies exploring the brain activity of two interacting persons [12], [16], [39]–[44]. It would be interesting to further understand aspects of joint creativity, how two or more individuals can generate complex and meaningful behavior together which is not a simple blend of their natural patterns.

## Supporting Information

File S1 **File includes Figures S1–S12 and Tables S1–S7.** Figure S1: Fourier analysis suggests a unique signature to each player and a universal region at which players have CC segments. Figure S2: Distribution of players' segments mean and variance values show no clear signature of players. Figure S3: Standard deviation distributions for skewness and kurtosis, for all players' leader segments, repeated players' leader segments and CC segments. Figure S4: (A) minimal jerk solution and sin(πx) function plotted together. (B) Amplitude distribution of the first and third Fourier components of players segments. Figure S5: CC segments show a characteristic relationship between frequency and velocity. Figure S6: Two main modes of playing are exemplified by segments sequence during a game in the frequency-maximal velocity plane. Figure S7: CC segments obeying a small dV-dT criterion lay in a universal region in the skewness-kurtosis plane. Figure S8: Ellipses of Blue leader, Red leader and CC segments of all games discussed in the main text. Figure S9: Histograms of Skewness and Kurtosis values of CC segments of Male-Male, Female-Female and Male-Female games. Figure S10: CC segments of novice-novice games have similar characteristics as CC segments from games with at least one expert. Figure S11: CC detector is independent on skewness and kurtosis values of the velocity segments. Figure S12: The correlation between leader and follower shows a peak at zero lag. Table S1: Correlation between segments velocity, frequency, skewness and kurtosis. Table S2: Percentage of differing games between red and blue leaders for each of the Fourier components. Table S3: Percentage of differing games comparing skewness and kurtosis values of every two players. Table S4: Percentage of differing games between red and blue leaders for skewness and kurtosis values. Table S5: Segments' mean skewness and kurtosis for Red and Blue handles. Table S6: Main CC segments characteristics are similar across experiments and gender. Table S7: Percentage of differing rounds leader vs. CC round for all players.
(DOCX)
