# Supplementary material for: Individuality and Togetherness in Joint Improvised Motion
Source: PLoS One. 2014 Feb 12;9(2):e87213. doi: 10.1371/journal.pone.0087213 (PMC3922750; doi:10.1371/journal.pone.0087213)
Supplement: File S1 — File includes Figures S1–S12 and Tables S1–S7. Figure S1: Fourier analysis suggests a unique signature to each player and a universal region at which players have CC segments. Figure S2: Distribution of players' segments mean and variance values show no clear signature of players. Figure S3: Standard deviation distributions for skewness and kurtosis, for all players' leader segments, repeated players' leader segments and CC segments. Figure S4: (A) minimal jerk solution and sin(πx) function plotted together. (B) Amplitude distribution of the first and third Fourier components of players segments. Figure S5: CC segments show a characteristic relationship between frequency and velocity. Figure S6: Two main modes of playing are exemplified by segments sequence during a game in the frequency-maximal velocity plane. Figure S7: CC segments obeying a small dV-dT criterion lay in a universal region in the skewness-kurtosis plane. Figure S8: Ellipses of Blue leader, Red leader and CC segments of all games discussed in the main text. Figure S9: Histograms of Skewness and Kurtosis values of CC segments of Male-Male, Female-Female and Male-Female games. Figure S10: CC segments of novice-novice games have similar characteristics as CC segments from games with at least one expert. Figure S11: CC detector is independent on skewness and kurtosis values of the velocity segments. Figure S12: The correlation between leader and follower shows a peak at zero lag. Table S1: Correlation between segments velocity, frequency, skewness and kurtosis. Table S2: Percentage of differing games between red and blue leaders for each of the Fourier components. Table S3: Percentage of differing games comparing skewness and kurtosis values of every two players. Table S4: Percentage of differing games between red and blue leaders for skewness and kurtosis values. Table S5: Segments' mean skewness and kurtosis for Red and Blue handles. Table S6: Main CC segments characteristics are similar across experi [file pone.0087213.s001.docx]

**Individuality and togetherness in joint improvised motion – Supplementary Information**

Yuval Hart*^,1,2^, Lior Noy*^,1,2^, Rinat Feniger-Schaal^3^, Avraham E Mayo^1,2^ and Uri Alon^1,2^

^1^Department of Molecular Cell Biology, Weizmann Institute of Science, Rehovot, Israel

^2^The Theatre Lab, Weizmann Institute of Science, Rehovot, Israel

^3^Graduate School of Creative Arts Therapies, The Center for the Study of Child Development, Haifa University, Haifa, Israel

## Segments’ skewness and kurtosis measures are weakly correlated with velocity and frequency

For each velocity segment (taken between two zero velocity points in the velocity trace of each player) we calculated the maximal velocity and frequency (defined as 1/(2*segment duration)).

Kurtosis and skewness were calculated on normalized segment traces, in which time was normalized between zero and one, and velocity was normalized by its integral over each segment$\int_{0}^{1} f\left( t \right)dt$. When correlating the kurtosis and skewness with the non-normalized maximal velocity and duration of each segment, we find weak correlations (Table S1). This indicates that the shape parameters- skewness and kurtosis- are not strongly affected by the maximal velocity and frequency of the motion at each segment.

In the table below, velocity is defined as the maximal velocity (the change in position of the handle through the time intervals of sampling), frequency is calculated as 1/(2*segment duration)), and skewness and kurtosis are calculated as in the main text, namely, $s=\frac{1}{V^{\frac{3}{2}}}\int_{0}^{1} \left( t-\mu\right)^{3}f\left( t \right)dt$ and$k=\frac{1}{V^{2}}\int_{0}^{1} \left( t-\mu\right)^{4}f\left( t \right)dt$ respectively (where µ is the center of mass and V is the variance of the segment).

Table S1: Correlation between segments velocity, frequency, skewness and kurtosis

|  | Velocity | Frequency | Skewness | Kurtosis |
| --- | --- | --- | --- | --- |
| Velocity | 1 | 0.31 | -0.27 | -0.04 |
| Frequency | 0.31 | 1 | 0.08 | 0.07 |
| Skewness | -0.27 | 0.08 | 1 | 0.05 |
| Kurtosis | -0.04 | 0.07 | 0.05 | 1 |

## Fourier analysis of segments shows individual signature and a universal CC region

In order to test the dependence of our results on the chosen features characteristics (meaning skewness and kurtosis of the velocity segments) we also decomposed the velocity segments by Fourier decomposition. Here we present analysis results for the first three components of this decomposition.

Players’ individuality is evident from the first and third components of the Fourier decomposition. The two players’ leader segments are different in more than 70% of the games for the first and third Fourier components (See Table S2). Furthermore, single players’ games show a unique signature in this frequency phase space, where single repeated players show unique medians (Fig. S1, Red and Orange lines). Variability between different players’ games as leaders is bigger by almost four-fold compared with the variability seen between games of the repeated single players that played many games (two players, playing each 16 and 8 games respectively, see Fig. S1A and S1C).

Moreover, we find that CC segments are localized in a universal region in phase space which is common to all games (see Fig. S1, Green line). CC segments median variability of the third Fourier component is on the order of variability of a single player segments between games (Fig. S1C). Median variability of the first Fourier component is about half of the variability of all players as leaders (Fig. S1A).

Table S2: Percentage of differing games between red and blue leaders for each of the Fourier components.

|  | 1^st^ Fourier component | 2^nd^ Fourier component | 3^rd^ Fourier component |
| --- | --- | --- | --- |
| Kolmogorov-Smirnov | 70% | 60% | 70% |
| Anderson-Darling | 80% | 60% | 80% |
| Cramer-von Mises | 73% | 50% | 83% |


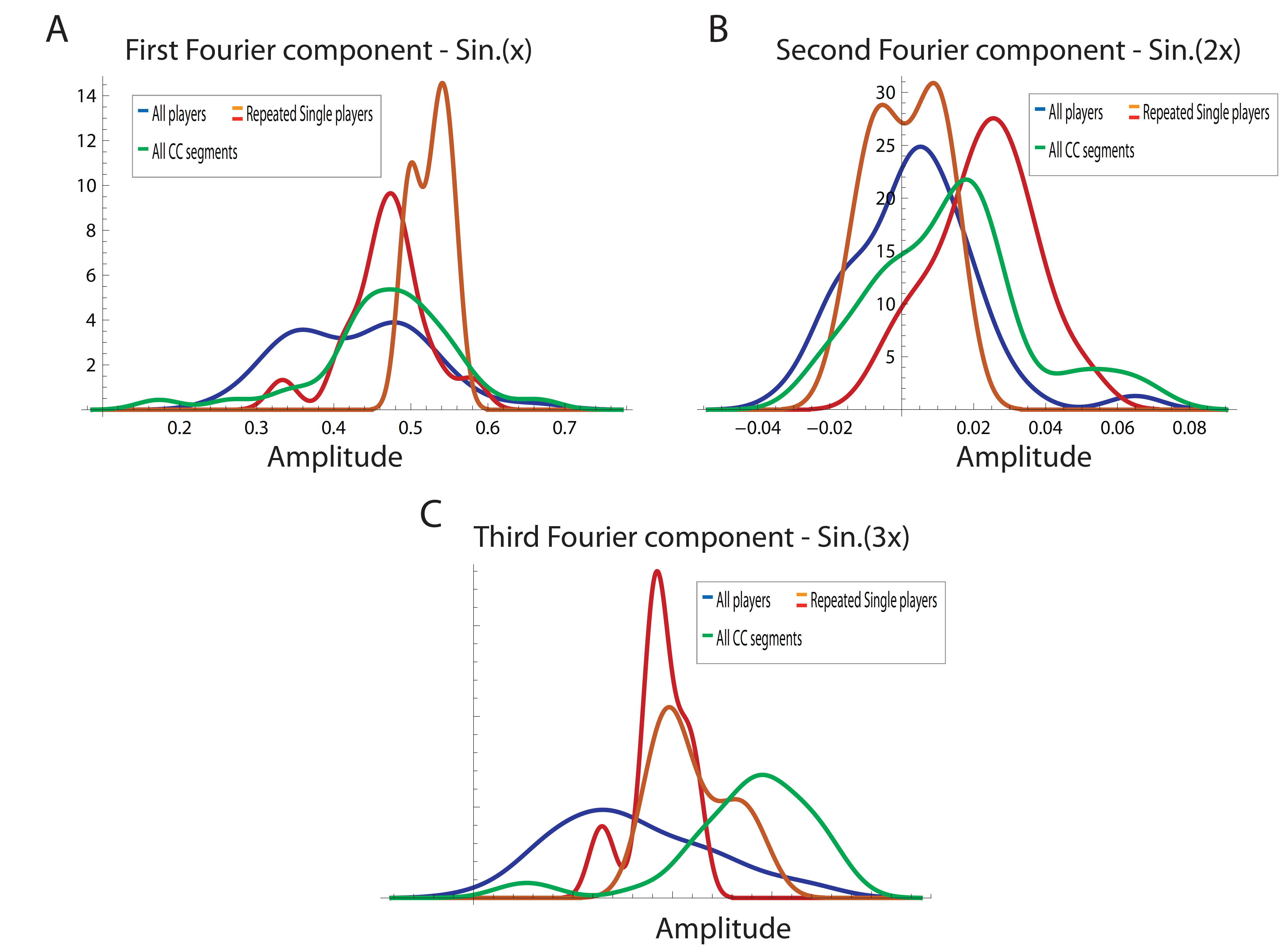


Figure S1: Fourier analysis suggests a unique signature to each player and a universal region at which players have CC segments. Shown are trimmed mean value distribution of all players (blue), two repeated players (red and orange) and CC segments (green) for the first Fourier component (A) second Fourier component (B) and the third Fourier component (C).

## Repeated players show large variability in their mean and variability values excluding differentiation based on these parameters

In the main text we analyze segments’ skewness and kurtosis values. In this section we show that players’ mean and variance values of the velocity segments do not confer similar information due to the high variability of a single player’s values.

Players’ leader segments mean values are different in 47% of the cases when comparing different players from all games (Mann-Whitney test, FDR set to 0.05, compare with 54% and 68% in skewness and kurtosis).

Analysis of the distribution of all players mean and variance values shows that a single repeated player shares the same variability between games as different individual players show (Fig. S2). Thus, a single player may produce segments with mean and variance values that are as spread as different players in different games show. Thus, although the variance of players at different games shows differentiability, there is no unique signature for players when considering their segments’ mean and variance values. In accordance, CC segments values also do not differ greatly in their distributions from the ‘all players’ distribution for both moments (see Fig. S2).


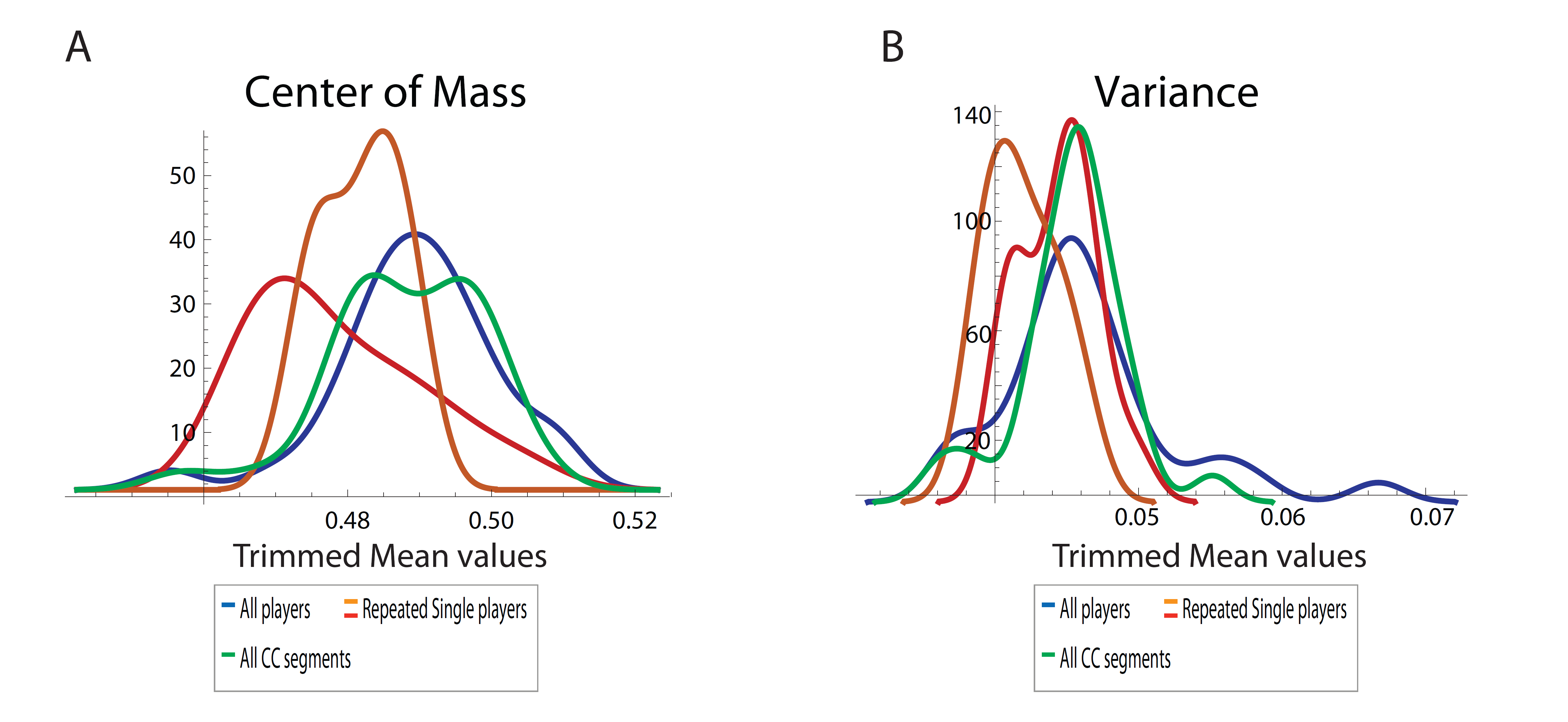


Figure S2: Distribution of players’ segments mean and variance values show no clear signature of players. (A) The distribution of mean trimmed average values (trim value was set to 0.2) of players’ leader segments (blue), two repeated single players’ leader segments (red and orange) and CC segments. Standard deviation of distributions is: 0.006, 0.008, 0.005, 0.007 respectively. (B) same as (A) for variance values. Standard deviation of distributions is: 0.0016, 0.0013, 0.0014, 0.0013 respectively.

## Players exhibit individual signatures in skewness-kurtosis motion space

Here we present full analysis of comparison between players’ signatures in terms of the following tests – t-test, Mann-Whitney, Kolmogorov-Smirnov, Anderson-Darling and Cramer-von Mises. Each player segment distributions are compared. In Table S3 we present test results for skewness and kurtosis values of all blue players’ leader segments in all games. In Table S4 we present test results for skewness and kurtosis values of red vs. blue players at their leader rounds in the same game. Two players are defined to have differing signatures if the p-value of the corresponding test was less than 0.05. FDR due to multiple hypothesis testing was set to 0.05 (1, 2).

Fig. S3 shows that players differ not only in their median values of segments values but also in the spread of their kurtosis and skewness values compared with each other and with comparison to the variability shown by the repeated single players who played several games (red and orange lines) or that of the CC segments (green line).

Table S3: Percentage of differing games comparing skewness and kurtosis values of every two players.

|  | Skewness | Kurtosis | Total (percent of at least one feature differing) |
| --- | --- | --- | --- |
| t-test | 54% | 58% | 79% |
| Mann-Whitney | 54% | 68% | 85% |
| Kolmogorov-Smirnov | 72% | 90% | 90% |
| Anderson-Darling | 79% | 92% | 92% |
| Cramer-von Mises | 78% | 90% | 90% |

Table S4: Percentage of differing games between red and blue leaders for skewness and kurtosis values.

|  | Skewness | Kurtosis | Total (percent of at least one feature differing) |
| --- | --- | --- | --- |
| t-test | 47% | 57% | 70% |
| Mann-Whitney | 60% | 60% | 80% |
| Kolmogorov-Smirnov | 63% | 83% | 87% |
| Anderson-Darling | 70% | 80% | 87% |
| Cramer-von Mises | 67% | 73% | 87% |


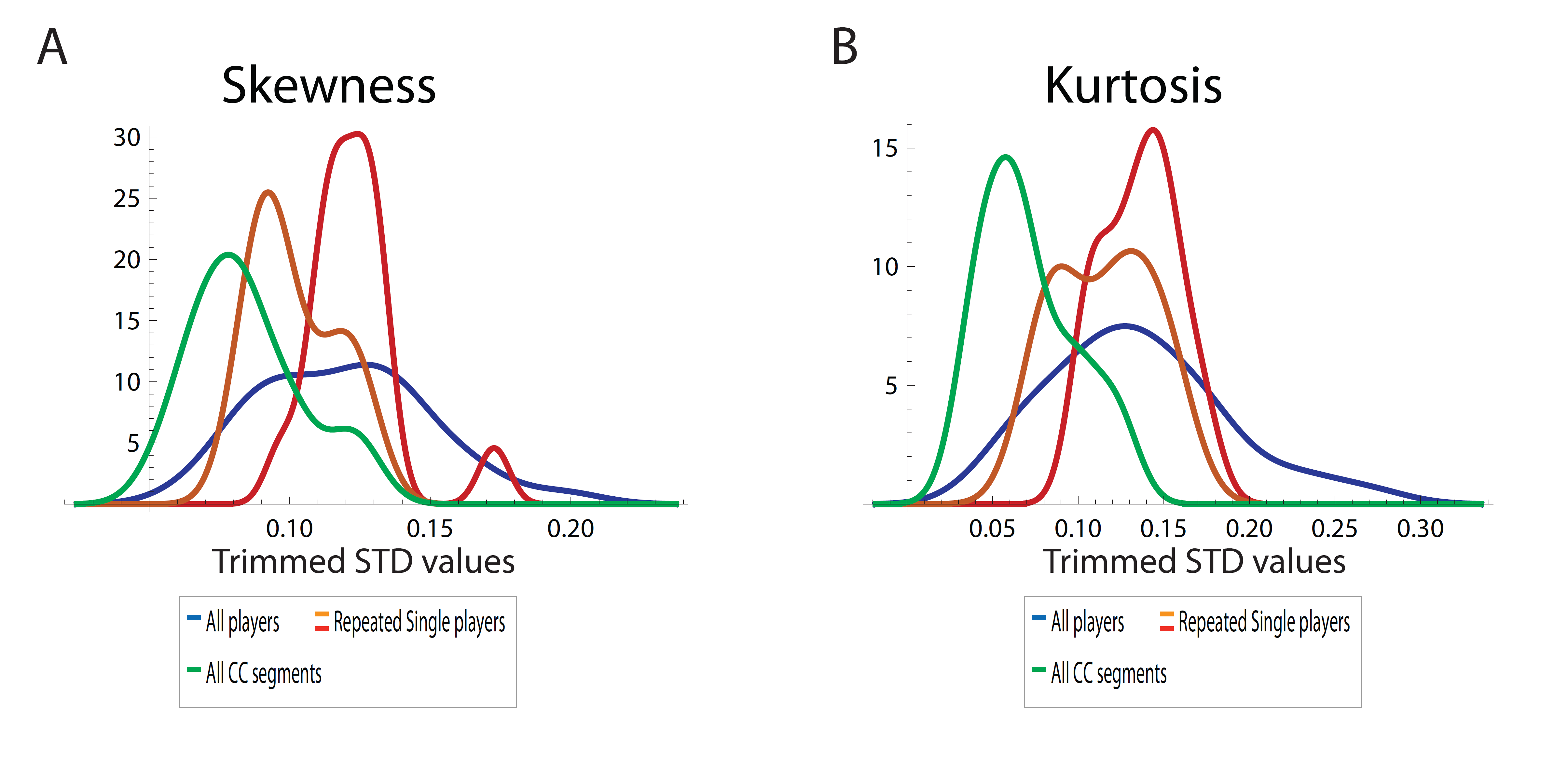


Figure S3: Standard deviation distributions for skewness (A) and kurtosis (B) displayed by all players’ leader segments (blue), repeated players’ leader segments (red and orange) and CC segments (green).

## Blue and red handles have similar segments characteristics

We verified that our results are not biased by a systematic difference between the motions produced by the two physical handles (red and blue). For this purpose we compared the segments of the players playing with the blue and the red handles in the six expert-expert games in our dataset with non-repeating players at the red handle (the games from Exp. 1 used in (3)). We compared all segments that were created by the red and blue player while playing the role of the leader in the expert-expert games.

We compared the Skewness (S) and the Kurtosis (K) of the segments, computed as described in the main text. We first removed from this analysis segments with outlier values, defined as S<=-1.5, S>1.5 or K>5. This cutoff removed 12 segments out of a dataset of 1171 leader segments in the six games (1%). We then compared the distributions of the Skewness and Kurtosis values of all segments from the red and blue handles. The results of this comparison appear in Table S5. A two-sample t-test showed no significant difference between the Skewness distributions of the red and blue players (t=0.19, N.S.), and similarly no significant difference between the Kurtosis distributions of the red and blue players (t=0.27, N.S.).

Table S5: Segments’ mean skewness and kurtosis for Red and Blue handles.

|  | Red handle | | Blue Handle | |
| --- | --- | --- | --- | --- |
|  | Mean | (SE) | Mean | (SE) |
| Skewness | 0.02 | (0.02) | 0.02 | (0.02) |
| Kurtosis | 2.04 | (0.02) | 2.03 | (0.03) |

## CC segments are enriched for smooth and periodic velocity trajectories

In this section we provide, for completeness, the analytical solution of the minimal jerk derived in (4) for periodic motion. We then show that CC segments are enriched with ‘sine-like’ segments relative to players’ leader segments.

Pioneering research (5, 6) on the motion people do when asked to move their hand from point A to point B suggested that people’s motion minimizes the change of acceleration a(t) throughout the movement. This was termed mean squared jerk and defined as:

1. $msj=\frac{1}{t_{2}-t_{1}}\int\frac{1}{2}\left( \dot{a} \right)^{2}dt$

For periodic motion the minimal movement solution value for x(t) is given by

1. $x\left( t \right)=x\left( 0 \right)+A\left( \frac{5}{2}\left( t^{2}-t^{4} \right)+t^{5} \right)$

Where A is the movement amplitude and time is rescaled to the total time of the movement. The velocity segment is given by the time derivative of x(t)

1. $v\left( t \right)=A(5t-10t^{3}+5t^{4})$

Plotting the smooth motion velocity described in Eq. (S3) against a half-sine wave indicates the two functions are highly similar (see Fig. S5A).

In figure S5B we present the amplitude of the first and third components in the Fourier decomposition of CC (togetherness) velocity segments compared with leader segments of players. Amplitudes of these symmetric components of CC segments are higher compared with leader segments – CC segments have mean amplitudes of 0.53 and 0.34 respectively, compared with amplitude values of 0.43 for the first Fourier component and 0.28 for the third Fourier component of the players’ leader segments. Thus, CC velocity segments may reflect a smoother and more predictable motion needed for joint improvisation.


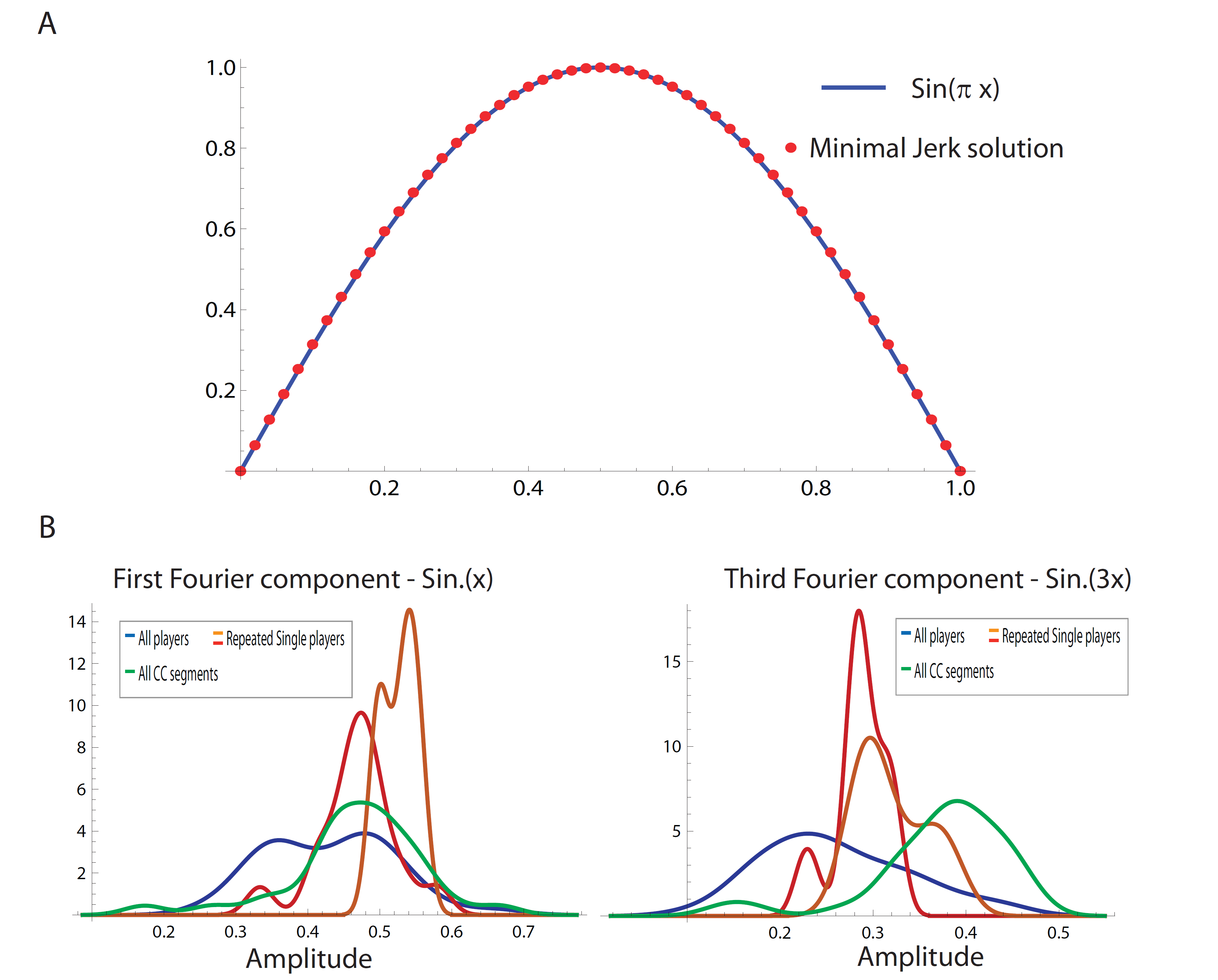


Figure S4: (A) minimal jerk solution and sin(πx) function plotted together (red dots and solid blue line respectively). (B) Amplitude distribution of the first and third Fourier components of players segments (blue – all players’ leader segments, orange and red – repeated single players’ leader segments and green – CC segments). Note that CC segments (green line) show higher enrichment of the symmetric Fourier components relative to the leader segments of all players (blue line).

## CC segments show a specific relation between frequency and maximal velocity

We further analyzed the frequency and maximal velocity properties of the CC segments.

For the analysis we chose segments in the highest density region in the frequency-maximal velocity plane (density values ranging between 0.7-1 from the maximal density value). We find that CC segments share a specific relationship between segment duration (frequency) and segment’s maximal velocity (Correlation coefficient of 0.92, See Fig. S6A). This relationship indicates a preferred length scale of 24.4 cm with the 95% confidence interval of 23-26 cm.

In contrast, when analyzing players’ leader segments with the same criterions we find a much weaker correlation of 0.35 between frequency and maximal velocity values. These segments’ length scale is found to be 35 cm with the 95% confidence interval spanning a large range of 29-43 cm (See Fig. S6B). Thus, CC segments are also characterized by a specific movement scheme in the frequency-velocity plane.


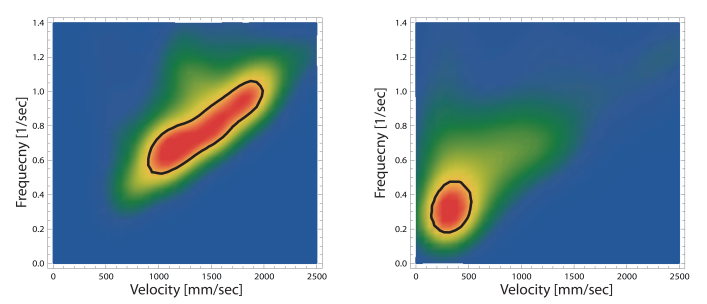


Figure S5: CC segments show a characteristic relationship between frequency and velocity. Panels show the density distribution of segments in the frequency-velocity plane for CC segments (left panel) and all players leader segments (right panel). Black contours represent the chosen area for calculating the characteristic length scale of each dataset (see section above).


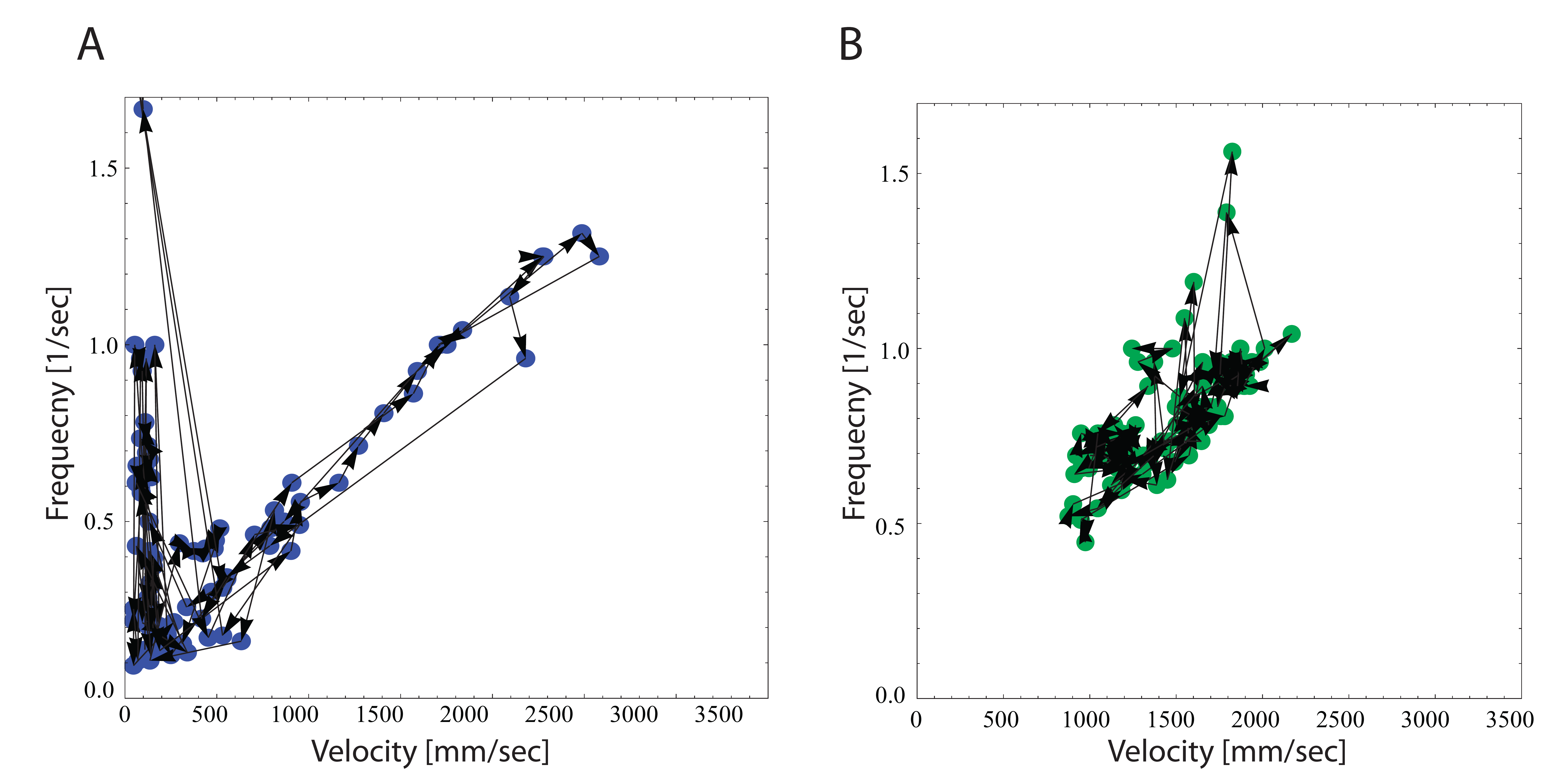


Figure S6: Two main modes of playing are exemplified by segments sequence during a game in the frequency-maximal velocity plane. Sequential segments are connected by an arrow, pointing from a previous segment to the next one. (A) An example of a blue player’s leader segments. Most segments show no clear relationship between frequency and velocity. Only a small fraction of the segments show a specific relationship between frequency and velocity. (B) An example of CC segments of a player at the rounds with no designated leader (joint improvisation). Most segments lie on a specific line in the frequency-velocity plane, indicating a certain relationship between the two motion features.

## An alternative definition for CC segments yields similar results to CC segments definition by no-jitter

In this section we show that the CC (togetherness) segments characteristics are similar also for a different criterion for defining a segment as CC.

In the main text, we marked segments as CC (togetherness) segments whenever both players did not exhibit any jitter (see Methods section in the main text). Here, we present a different criterion for marking CC segments: Segments are defined as CC if the rms difference between the segments of the two players (denoted dV) is smaller than 35% and the time difference between the duration of the two segments (denoted dT) is smaller than 80msec. This definition assumes that CC segments are those where the two players create velocity trajectories that are similar in shape.

In total, there are 5896 CC Segments chosen by following the criterion of small dV-dT (16.5% of all segments). 2603 of these segments (44.2%) were also identified as CC segments by using the no-jitter criterion.

Analyzing these segments for their skewness and kurtosis characteristics shows similar results to the results obtained using the no-jitter criterion. Skewness and kurtosis values of the CC segments are shared among different games (see Fig. S7) and lie in the same universal region as those for the no-jitter criterion. Their distribution among games is 1.6 fold narrower than players’ leader games and similar to repeated single players games in kurtosis values (see Fig. S7). Moreover, these segments show low kurtosis values and near zero skewness, suggesting these segments are similar to the minimal jerk solution (see main text and section above) and are smooth and predictable.


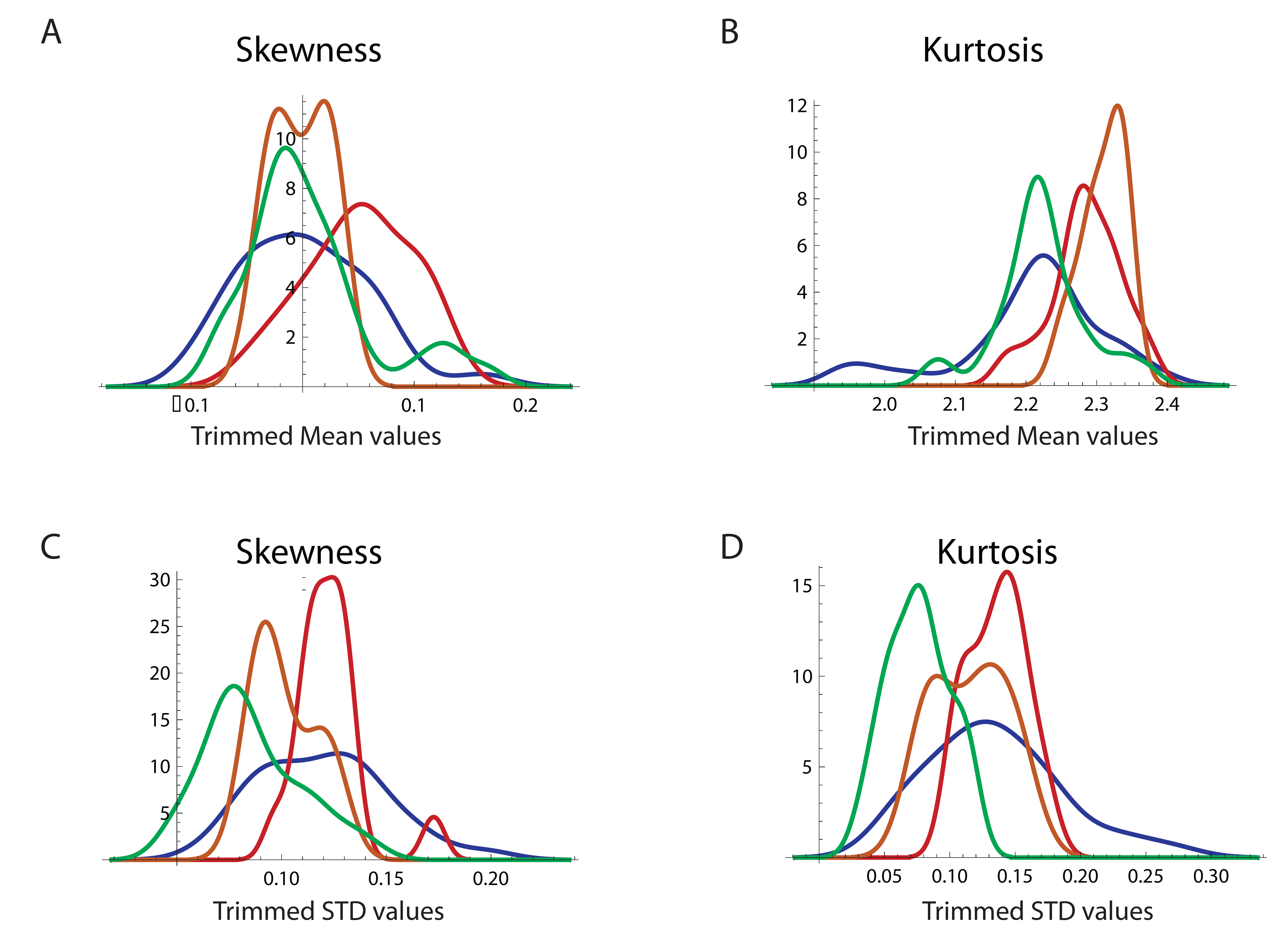


Figure S7: CC segments obeying a small dV-dT criterion lay in a universal region in the skewness-kurtosis plane (A) The distribution of skewness trimmed average values (trim value was set to 0.2) of players’ leader segments (blue), two repeated single players’ leader segments (red and orange) and CC segments chosen by a small dV-dT criterion. Standard deviation of distributions is: 0.04, 0.03, 0.02, 0.03 respectively. (B) same as (A) for kurtosis values. Standard deviation of distributions is: 0.05, 0.03, 0.02, 0.03 respectively. (C) The distribution of skewness trimmed standard deviation values (trim value was set to 0.2) of players’ leader segments (blue), two repeated single players’ leader segments (red and orange) and CC segments chosen by a small dV-dT criterion. (D) same as (C) for kurtosis values

## 9. All games panels of red/blue/CC ellipses


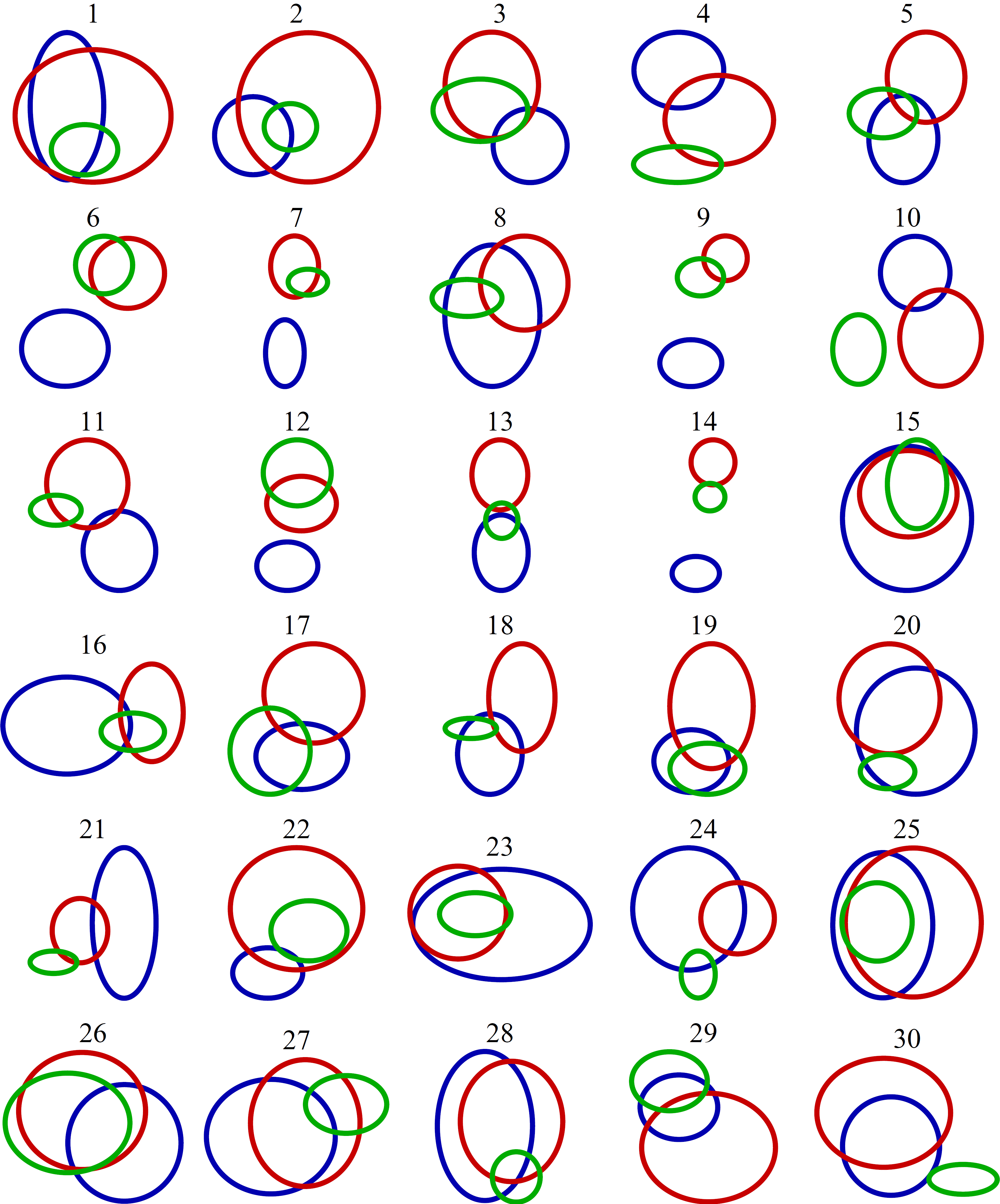


Figure S8: Ellipses of Blue leader, Red leader and CC segments of all games discussed in the main text. Ellipse center is the trimmed average (80%) of all segments. Ellipse axes represent the trimmed standard deviation (80%) of the segments in each axis (skewness and kurtosis). For clarity of presentation game panels are not shown on the same scales. Note that in many cases the green (CC) ellipse does not reside in an intermediate region between the individual red and blue ellipses.

## Male-Male, Female-Female and Female-Male games show similar motion characteristics and differ in CC ratio values

Table S6 below compares mean and standard deviation of skewness and kurtosis values of CC segments and CC ratio between the games of Exp.1, Exp.2, Exp.3 and also values of games where male-male participants played, female-female participants played and female-male participants players.

Table S6: Main CC segments characteristics are similar across experiments and gender

|  | Exp. 1 (Expert-Expert) | Exp. 2 (Expert-Novice, Males) | Exp. 3 (Expert-Novice, Females) | Male-Male | Female-Female | Female-Male |
| --- | --- | --- | --- | --- | --- | --- |
| Skewness mean | -0.02 | 0.01 | -0.03 | 0.01 | -0.03 | -0.03 |
| Skewness standard deviation | 0.15 | 0.17 | 0.14 | 0.17 | 0.14 | 0.13 |
| Kurtosis mean | 2.23 | 2.24 | 2.26 | 2.26 | 2.25 | 2.23 |
| Kurtosis standard deviation | 0.13 | 0.17 | 0.14 | 0.18 | 0.13 | 0.12 |
| CC ratio mean | 0.12 | 0.2 | 0.13 | 0.21 | 0.11 | 0.22 |
| CC ratio standard deviation | 0.12 | 0.12 | 0.04 | 0.12 | 0.05 | 0.15 |


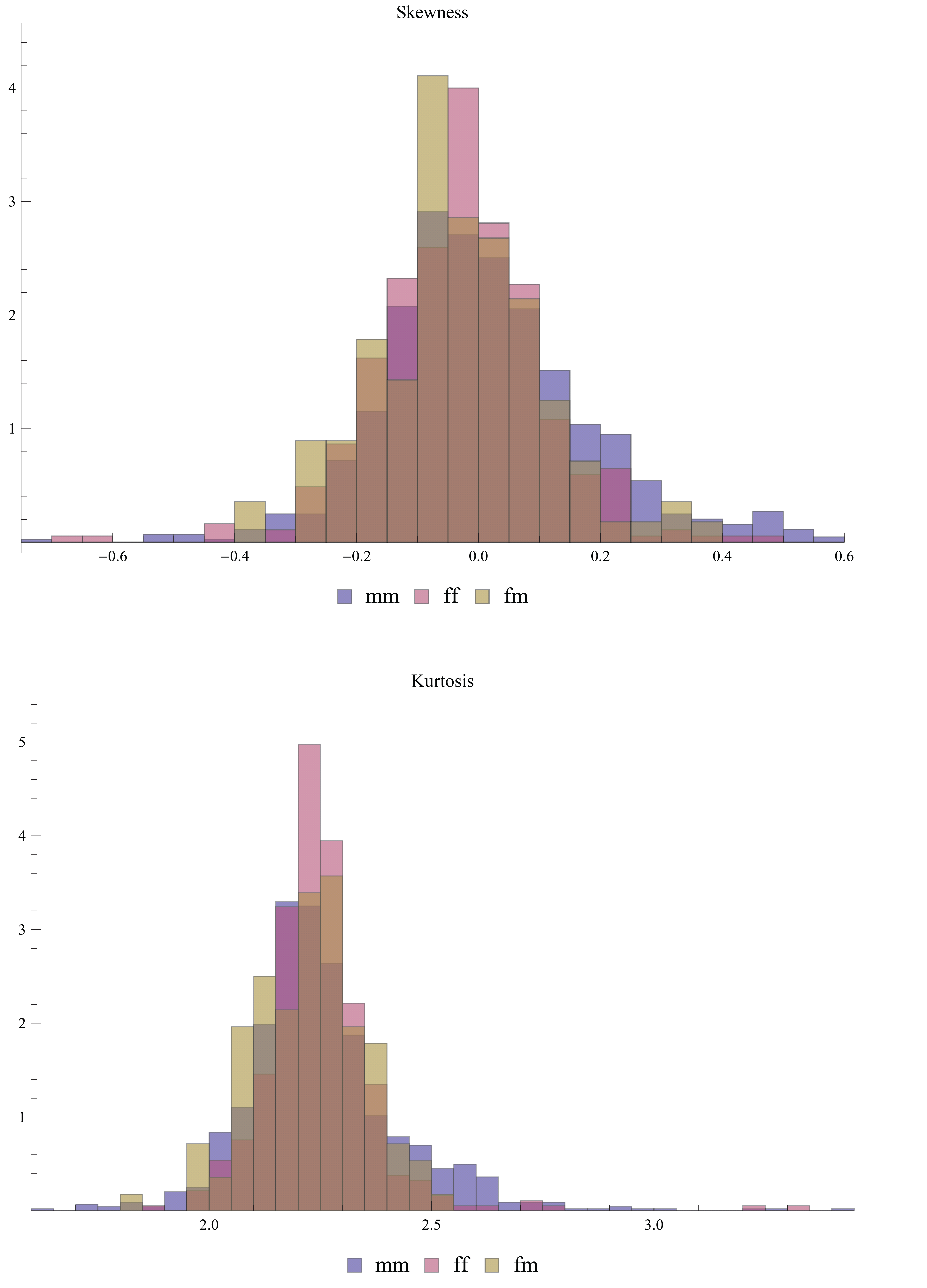


Figure S9: Histograms of Skewness and Kurtosis values of CC segments of Male-Male, Female-Female and Male-Female games.

## 11. Novice-Novice games show less CC segments, these segments lay in the same region as found in Expert-Novice, Expert-Expert games

We further report analysis results of eight games where both players were novices (see Ref (3) for experiment details). These novice-novice games show a much decreased CC ratio of 6±4% compared with the 17% CC ratio found in the experiments with at least one expert. While the possibility for a CC segment to occur is much smaller, analysis of the CC skewness and kurtosis values of novice-novice games indicates that it also lies in the universal CC region found in the main text for games with at least one expert. Skewness and kurtosis mean and standard deviation values are - 0.01±0.18 and 2.4±0.23. It should be noted that due to the scarceness of CC segments in novice-novice games, statistics values are not well sampled. See Figure S10 for comparison between CC values of novice-novice games and CC values of games with at least one expert.

Figure S10: CC segments of novice-novice games have similar characteristics as CC segments from games with at least one expert. Cross center represents trimmed mean and cross axes are trimmed STE values in the skewness-kurtosis plane. Orange cross represents all novice-novice CC segments (joined across eight games). Green crosses are the CC segments in games with at least one expert. Blue crosses are segments of blue leaders across the 30 games with at least one expert.

## 12. CC detector recognizes CC regardless of skewness and kurtosis values

We wanted to check whether the algorithm for detecting co-confident motion used in the main text is dependent on specific skewness and kurtosis values. In particular we wished to check that the CC detection algorithm does not recognize only segments with the specific skewness-kurtosis values found for the universal region. We thus constructed four pairs of artificial, computer generated velocity traces which are composed from a repeated segment with a distinct skewness and kurtosis values. Each pair has one velocity trace added with a small noise term (a slow frequency fluctuating sine with a noise term, where the noise frequency was 10 fold reduced compared to the velocity trace frequency). Skewness-Kurtosis values of each pair were: (0.28, 1.66), (-0.08, 1.96), (0.73, 2.91), (-0.68, 2.75). In all pairs, the detector identified co-confident (CC) segments, indicating that detection is not dependent on skewness and kurtosis values
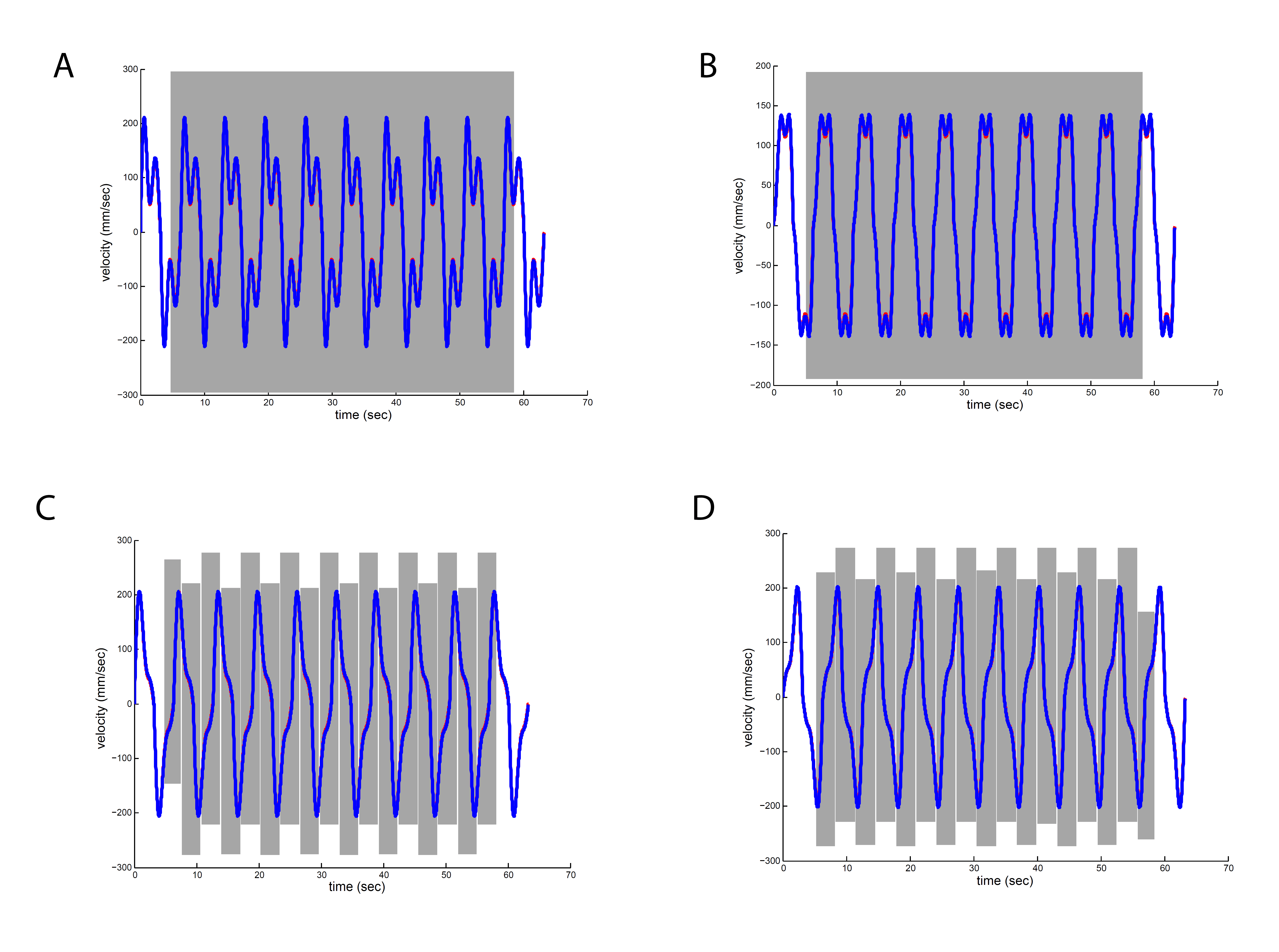
Figure S11: CC detector is independent on skewness and kurtosis values of the velocity segments. Four different pairs of (skewness, kurtosis) segments of velocity traces were generated artificially by a computer and detected by the CC detection algorithm. The skewness-kurtosis values were (0.28, 1.66), (-0.08, 1.96), (0.73, 2.91), (-0.68, 2.75). The two simulated velocity segments differ by a small noise term of sin(0.1 w t) and are marked by blue and red. Gray regions are times identified by the detection algorithm as a co-confident motion of the two players.

## 13. Players CC motion characteristics are different from their motion characteristics as leaders

In this section we present comparison of leader vs. CC motion per player, in order to check that indeed players change their signature when doing CC motion in the togetherness periods.

We used t-test and Mann-Whitney test in order to compare between skewness and kurtosis values of the two rounds. Results are shown at Table S7 below. FDR was set to be 0.05 to control for multiple testing (2).

Table S7: Percentage of differing rounds leader vs. CC round for all players.

|  | Skewness | Kurtosis | Total (differing at least in one feature) |
| --- | --- | --- | --- |
| *t*-test | 33% | 43% | 50% |
| Mann-Whitney | 37% | 47% | 60% |

## 14. Leader-Follower velocity traces show zero lag between them

A reasonable hypothesis might be that people when acting as followers merely lag behind the leader’s movement. We find that the correlation function between the leader and follower’s velocity trajectories has a peak at zero lag. This could be explained by the jitter motion found in our experiments, were the follower player constantly corrects the motion to fit to the leader’s current motion. Thus, the follower weaves around the leader’s motion (with a 2-3 Hz period), and thus the follower is sometimes ahead and sometime behind the leader (see Fig. S12).


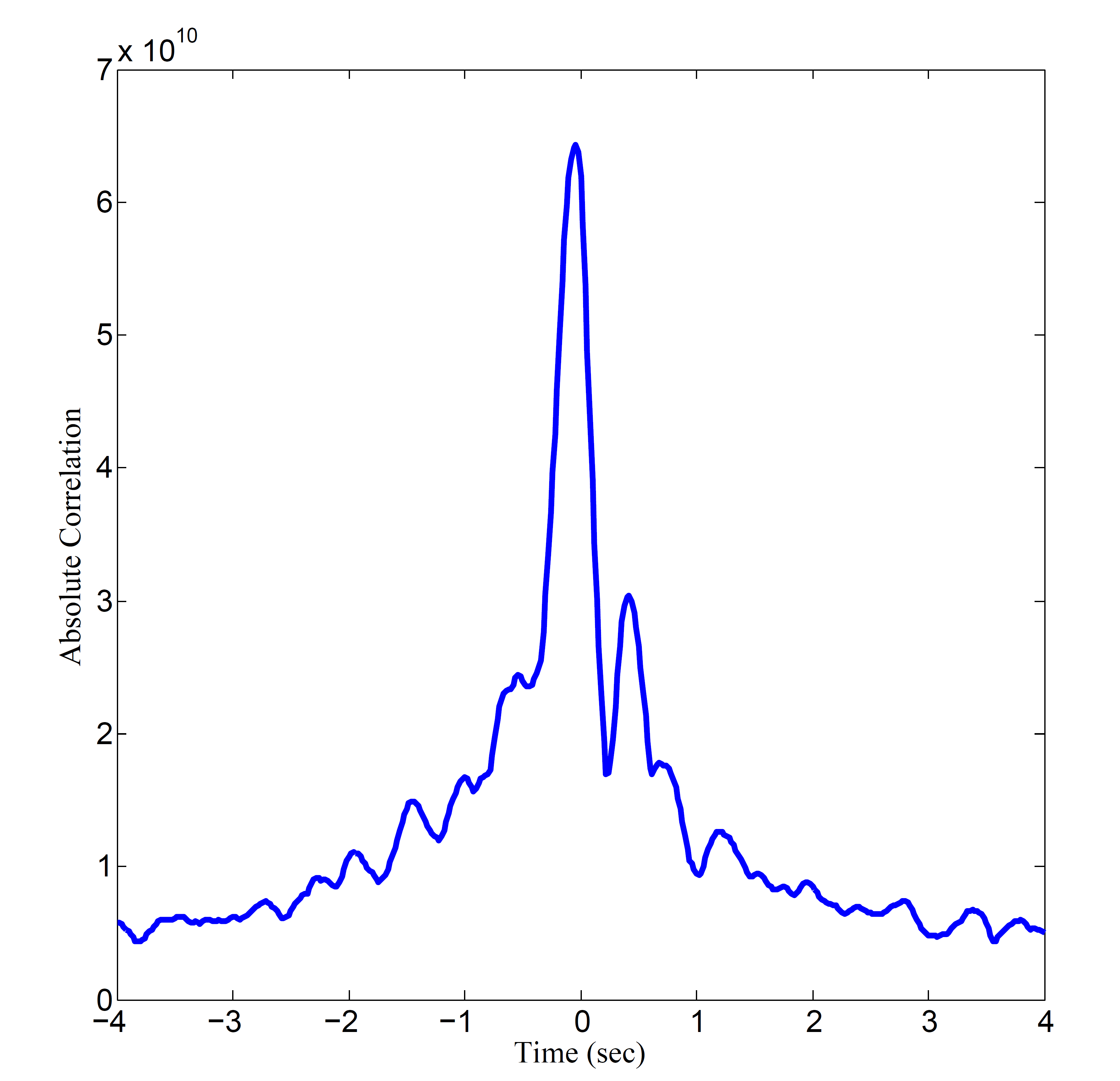


Figure S12: The correlation between leader and follower (sum over all games of all absolute correlation function) shows a peak at zero lag.

## 15. Possible implications of our results on human interaction

To the extent that the mirror game can serve as a model of interaction between two people, the present findings might relate to mode of behavior that helps promote togetherness. In order to increase the chance of togetherness, people may need to leave their natural idiosyncratic tendencies and adopt special, inviting modes of elementary behaviors. For example, a person who aims to actively listen to another, as in counseling and therapy, makes certain sounds and movements that invite the other to talk and to deepen listening– attending to the others body postures, making listening sounds, reflecting content, etc. (7, 8). It is important to remember that this statement relates to elementary behavior modes - the letters, not the words. Arbitrarily complex content can be made with this elementary behavior.

## References

1. Storey JD (2002) A direct approach to false discovery rates. *Journal of the Royal Statistical Society: Series B (Statistical Methodology)* 64:479–498.

2. Benjamini Y, Hochberg Y (1995) Controlling the False Discovery Rate: A Practical and Powerful Approach to Multiple Testing. *Journal of the Royal Statistical Society Series B (Methodological)* 57:289–300.

3. Noy L, Dekel E, Alon U (2011) The Mirror Game as a Paradigm for Studying the Dynamics of Two People Improvising Motion Together. *Proc Natl Acad Sci USA* 108:20947–20952.

4. Hogan N, Sternad D (2007) On rhythmic and discrete movements: reflections, definitions and implications for motor control. *Exp Brain Res* 181:13–30.

5. Flash T, Hogan N (1985) The coordination of arm movements: an experimentally confirmed mathematical model. *J Neurosci* 5:1688–1703.

6. Hogan N, Flash T (1987) Moving gracefully: quantitative theories of motor coordination. *Trends in Neurosciences* 10:170–174.

7. Rogers CR (1951) *Client-centered therapy: its current practice, implications and theory* (Constable, London).

8. Miller WR, Rollnick S (2013) *Motivational interviewing: helping people change* (Guilford Press, New York, NY).
